# Nutrition and Avoidance Diets in Children With Food Allergy

**DOI:** 10.3389/fped.2020.00518

**Published:** 2020-09-04

**Authors:** Domenico Corica, Tommaso Aversa, Lucia Caminiti, Fortunato Lombardo, Malgorzata Wasniewska, Giovanni Battista Pajno

**Affiliations:** Department of Human Pathology of Adulthood and Childhood G. Barresi, University of Messina, Messina, Italy

**Keywords:** early-introduction, elimination diet, food allergen, non-IgE-mediated food allergy, tolerance, immunotherapy

## Abstract

Food allergy (FA) is a significant health issue which considerably influences the quality of life of both children and their family. The increasing prevalence of FA, documented in the last 3 decades, has led to the reassessment of FA prevention strategies and particularly to giving up the approach based on delaying the introduction of potential food allergens. Several observational and interventional studies demonstrated a potential effectiveness of the early food introduction strategy in FA prevention, although strong evidence from randomized controlled trials are lacking and, sometimes, contrasting. The current approach to FA is mainly based on avoidance diet and the use of rescue medications in case of allergic reaction, although active allergen immunotherapy has recently become an increasingly important therapeutic strategy to approach IgE-mediated FA, potentially able to induce improvement through desensitization to a specific food. This review provides an overview on the historical evolution of recommendations about FA and on evidence published in the last 15 years on nutritional intervention strategy, i.e., early introduction of allergen or avoidance diet, in the prevention and management of IgE-mediated and non-IgE-mediated FA in children.

## Introduction

Food allergy (FA) is a significant health issue with an increasing prevalence in the last 30 years, affecting up to 6–8% of children worldwide (1–4) and up to 10% in high-income countries (5). FA presents a very heterogeneous clinical spectrum, which varies from mild and self-limited reactions to severe anaphylaxis, and it is often encumbered by a significant reduction in the quality of life (QoL) of both patients and their family (6, 7). Therefore, the development and improvement of FA treatment has become a public health priority, mainly in cases of potentially life-threatening reactions (8).

FA can be categorized in (a) IgE-mediated allergic reactions, characterized by an acute onset of symptoms, usually within a few minutes or a few hours after exposure to food antigen; (b) non-IgE mediated reactions, where there is a delayed onset of symptoms, mainly gastrointestinal ones; and (c) mixed IgE and non-IgE mediated food allergy.

The natural history of FA is usually characterized by presentation in the early stages of allergic march along with atopic dermatitis, sometimes associated to more severe allergic reactions, and by spontaneous tolerance for food allergens such as cow's milk (CM) and hen's egg (HE) within early school-age years. However, in some cases, FA persists over time with a negative impact on the QoL of patients and their family (9). The current approach to FA is mainly based on avoidance diet and the use of rescue medications in the case of an allergic reaction. Alternatively, active allergen immunotherapy (AIT), the only available treatment able to potentially induce a resolution of FA, could be performed in selected patients followed up in highly specialized centers (8).

The increased prevalence of FA has led to the reassessment of prevention strategies and, in particular, to give up the approach based on delaying the introduction of potential food allergens in infants at high risk of atopy, which results in unsuccessful FA prevention and potentially negatively affects FA natural history. Therefore, in the last decade, several studies have evaluated an early food introduction approach for the prevention of FA, demonstrating the potential effectiveness of this strategy in FA prevention. This review focuses on the most recent studies, published in the last 15 years, that investigated nutrition intervention strategy, i.e., early introduction of allergen or avoidance diet, in the prevention and management of IgE-mediated and non-IgE-mediated FA in children. The possible role of vitamin D, pro- or prebiotics, or short-chain fatty acids as nutritional strategies against FA will not be discussed in this review. Research was carried out through MEDLINE via PubMed (http://www.ncbi.nlm.nih.gov/pubmed/), Embase, CINAHL, and Cochrane Library, based on the combinations of two or more of the following keywords: (Food allergy) AND (nutrition OR diet OR prevention OR avoidance diet OR early introduction) AND (milk OR egg OR peanut OR fish OR cereal OR tree nut) AND (tolerance OR immunotherapy) AND (IgE-mediated OR non-IgE-mediated) AND (children OR pediatrics). Research included articles written in English belonging to the categories of clinical trial, observational study, meta-analysis, multicenter study, randomized controlled trial, or review.

## Historical Overview

Over the last 40 years, the prevention and treatment of FA has been extensively investigated due to its high social and healthcare cost (10).

Between the late 1900's and early 2000's, several recommendations indicated the delay of dietary allergen introductions in infants, based on the hypothesis that exposure to solid foods in early infancy could increase the risk for allergic sensitization. In 1974, the *Present-Day Practice in Infant Feeding* guidelines (11) discouraged both the early introduction of cereals and other solid food into babies' diet before 4 months of age. This recommendation was based on the assumption that an early and increased incidence of celiac disease in Britain, between the 1960's and early 1970's, had been related to precocious introduction of gluten. This assumption was supported by a subsequent decline of incidence, between 1974 and 1979, that had been observed after the changes in infant feeding practices (12). By the late 1990's, the World Health Organization (WHO) recommended a further delay of first exposure to solid foods to 6 months of age, and to delay eggs and peanut introduction to 10 months and 3 years of age, respectively (13). In the early 2000's, similar indications were proposed by American guidelines that recommended the introduction of dairy products and egg at 12 and 24 months of age, respectively, and peanut, tree nut, fish, and seafood at 36 months of age, in particular in infants with a family history of atopy in first-degree relatives (14, 15).

These recommendations, based on insufficient evidence, did not determine a reduction in FA incidence, as expected, but, on the contrary, probably promoted a further increase of FA prevalence in association with genetic, epigenetic, and environmental factors. The most relevant theories trying to explain the role played by environmental factors are: the *hygiene hypothesis*, sustaining the implication of early-life microbial exposure to antigens in the regulation of immune response and in the prevention of allergic diseases; the *vitamin D hypothesis*, supporting the action of vitamin D deficiency in furthering the development of FA; and the *dual-barrier hypothesis*, suggesting the potential role of transcutaneous early exposure to food allergens in FA pathogenesis (16).

Consistent with the dual-barrier hypothesis, several studies demonstrated the close relationship between disrupted skin barrier and FA in infants. Transcutaneous sensitization to peanut protein in children with inflamed skin through topical peanut-oil emollient application (17), as well as other environmental exposure, have been associated with higher risk of peanut allergy (18, 19). Kelleher et al. documented that neonatal skin barrier alterations predicted FA at 2 years of age (20). These results were supported by studies on murine models and filaggrin mutations. Noti et al. demonstrated in murine models that transcutaneous exposure to ovalbumin or peanut through an atopic dermatitis-like skin lesion promoted immunological mechanisms related to FA development, specifically by an increase of Th2 cytokine response, antigen-specific serum IgE levels, and localization of mast cells in the intestine (21). Strid et al. concluded with similar results, highlighting that epicutaneous exposure to peanut protein selectively promoted Th2 immune response and prevented the induction of oral tolerance in murine models (22). Patients with filaggrin loss-of-function mutations were more likely to develop sensitization to food allergens and FA (23, 24).

Moreover, maternal solid allergen-free diet during pregnancy and lactation in the prevention of FA in infants has been confirmed ineffective (18, 25–28), and on the contrary, it seems to be able to induce sensitization (29).

Evidence and results of other observational studies (30, 31) definitively clarified that early-life avoidance strategy to food allergens does not prevent FA; instead, it could contribute to promote the increasing prevalence of FA in association with genetic, epigenetic, and environmental factors. Accordingly, American guidelines recommended the introduction of complementary foods between 4 and 6 months of age (32, 33). However, guidelines specifying the timing of introduction of potential allergenic solid foods are not available (34), except for peanut (35).

## Current Status for IgE-Mediated Food Allergy

An increasing number of observational and interventional studies have been carried out to assess the timing of first exposure to allergens and to evaluate the role of the early introduction of food allergens in the prevention of FA. Even though observational studies support that early allergen ingestion can be effective in FA prevention, strong evidence from randomized controlled trials (RCT) are lacking.

### Milk

Observational studies suggested that avoidance diet and the delayed introduction of CM proteins did not prevent CM allergy, and on the contrary, early introduction could promote tolerance. A large prospective birth cohort study evaluated the association between age of CM and other foods' first introduction and infant atopic manifestations and sensitization (specific IgE levels) in the first 2 years of life (36). Authors demonstrated that the delayed introduction of CM proteins and other solid foods was associated with an increased risk for atopic manifestations, such as eczema and recurrent wheeze (36). In another large-scale population-based prospective study, a significantly lower frequency of IgE-mediated CM allergy was documented in infants precociously exposed to CM proteins (within 14 days of life) compared to delayed introduction (between 105 and 194 days of life), allowing authors to conclude that early exposure to CM proteins, in association to breastfeeding, might promote tolerance (37). Consistently, in a double-blind, randomized controlled trial (DBRCT) involving children with a family history of allergic diseases, partially hydrolyzed whey-dominant formula supplemented with a specific oligosaccharide mixture was ineffective to prevent eczema at 12 months of life compared to the standard CM formula (38).

The *Enquiring About Tolerance* (EAT) trial is the only available interventional study evaluating the early introduction of CM proteins (39). In this randomized controlled trial (RCT), CM proteins (yogurt) were the first administered allergen to 3-month-old breast-fed infants, followed by other six allergens (cooked HE, peanut, white-fish, sesame, wheat) (39). Even though the low rate of per-protocol adherence to allergen assumption in the early-introduction group influenced study result interpretation as a whole, the adherence rate for CM was acceptable (85.2%). In the per-protocol analysis, CM allergy rate was not significantly different between groups, although it was lower in the early-introduction group (3–6 months of age) than in the standard-introduction group (after 6 months of age). Similarly, in the intention-to-treat analysis, at 3 years of age, CM allergy rate as well as allergy rate to other allergens was not significantly lower in the early-introduction group compared to the standard-introduction group (39).

### Egg

Available data about the efficacy of HE early introduction to prevent sensitization and allergy are contrasting (34). A large cross-sectional study documented a higher risk to develop HE allergy in children who underwent delayed introduction of cooked egg (≥10 months of age) than those who received HE at 4–6 months of age (40). In accordance with this result, in the per-protocol analysis of EAT trial a significantly lower prevalence of HE allergy was documented in early-introduction group (rate of adherence 43.1%) than in controls, and the consumption of at least 4 grams per week of egg protein was associated with a significantly lower prevalence of egg allergy than less consumption (39) (Table 1).

**Table 1:** Overview of RCTs evaluated with early introduction of hen's egg.

**Trial**	**Study design**	**Primary outcome**	**Active group selection criteria**	**Active group/controls (n) included in the primary analysis**	**Allergen formulation (vehicle)**	**Intervention (control)**	**Limitations**
EAT (39)	RCT	Prevalence of challenge-proven FA to HE or to other 5 foods in early-introduction group between 1 year and 3 years of age	3-month-old exclusively breast-fed infants	652/651	Whole hard-boiled egg (not specified)	Early-introduction group (3–6 months of age): 4 g of egg protein/week (equivalent to 2 g of egg-white protein) (Standard-introduction group: introduced the same egg proteins amount after 6 month of age)	Low rate of per-protocol adherence in the early-introduction group.
PETIT (41)	DBRCT	Prevalence of HE allergy confirmed by OFC at 12 months of age	4–5-month-old infants with AD, never orally exposed before to HE	60/61	Heated egg powder (squash, Japanese pumpkins)	Stepwise introduction of allergen From 6 to 9 months of age: 25 mg of egg proteins/daily; From 9 to 12 months of age: 125 mg of egg proteins/daily (Placebo from 6 to 12 months of age)	The study was early stopped because of a large group difference at the planned interim analysis.
STAR (42)	DBRCT	Diagnosis of IgE-mediated HE allergy by SPT and OFC at 12 months of age	4-month-old infants with moderate-to-severe AD, never orally exposed before to HE	42/35	Pasteurized raw whole egg powder (infant rice cereal)	From 4 to 8 months of age: 0.9 g of egg protein/daily From 8 months of age: medically supervised cooked egg exposure (Placebo from 4 to 8 months of age)	- Recruitment was early stopped for logistic reason, without reaching the sample size originally estimated. - 31% of active group patients stopped egg powder ingestion due to allergic reactions.
HEAP (43)	RCT	Defined HE sensitization by sIgE at 12 months of age	4–6-month-old infants from general population with HE sIgE levels <0.35 kUA/L	156/142	Pasteurized egg white powder (solid baby food)	From recruitment to 12 months of age, 3 times/week: - 0.8 g in the first week - 1.6 g in the second week - 2.5 g from the third week of intervention to 12 months of age. (Placebo from recruitment to 12 months of age)	- Recruitment was early stopped for allergic reaction in active group at first exposure to allergen.
STEP (44)	RCT	Diagnosis of IgE-mediated HE allergy and sensitization by OFC and SPT, respectively, at age 12 months	4–6.5-month-old infants with atopic mothers and without history of allergic disease or previous egg ingestion	407/413	Pasteurized raw whole egg powder (carrot, pineapple, and rice powders)	From recruitment to 10 months of age: 0.4 g egg protein/daily From 10 months of age: cooked egg and egg-containing foods were included in diet of both groups. (Placebo from recruitment to 10 months of age)	- Inability to reach the planned sample size. - Relatively small amount of dietary egg.
BEAT (45)	DBRCT	Prevalence of HE sensitization confirmed by SPT at 12 months of age	4–6-month-old infants no-sensitized to HE with a family history for allergy (at least one first-degree relative with allergic diseases)	165/154	Pasteurized whole egg powder (not specified)	From recruitment to 8 months of age: 350 mg egg protein/daily From 8 months of age: liberalized diet in active group and controls. (Placebo from recruitment to 8 months of age)	- Relatively small amount of dietary egg. - Impossibility to challenge all infants with possible egg allergy at 12 months of age.

*FA, Food allergy; HE, hen's egg; DBRCT, double-blind randomized controlled trial; RCT, randomized controlled trial; sIgE, specific IgE; EAT, Enquiring About Tolerance; PETIT, Prevention of Egg Allergy with Tiny Amount Intake; STAR, Solid Timing for Allergy Research; HEAP, Hen's Egg Allergy Prevention; STEP, Study Starting Time of Egg Protein; BEAT, Beating Egg Allergy Trial; OFC, oral food challenge; AD, atopic dermatitis; SPT, skin prick test*.

The results of *Prevention of Egg Allergy with Tiny Amount Intake* (PETIT) DBRCT also supported an early introduction of allergen for HE allergy prevention, although findings should be interpreted taking into consideration the considerable drop-out (41). More specifically, a cohort of 4–5-month-old infants with eczema, never orally exposed to HE before, were enrolled to undergo a stepwise introduction of egg protein (in the form of heated egg powder) or placebo from 6 months of age (41) (Table 1). At 12 months, a significant lower prevalence of HE allergy in the active group compared to the placebo control group has been documented. Moreover, authors reported a significantly lower level of ovomucoid-specific IgE and a higher concentration of ovomucoid-specific IgG1, IgG4, and IgA in the active group compared to placebo group (41).

Conversely, the *Solid Timing for Allergy Research* (STAR), the *Hen's Egg Allergy Prevention* (HEAP), the *Study Starting Time of Egg Protein* (STEP), and the *Beating Egg Allergy Trial* (BEAT) trials did not demonstrate that early consumption of HE was able to prevent HE sensitization and allergy (42–45). The STAR DBRCT evaluated the IgE-mediated HE allergy rate in infants with moderate-to-severe eczema daily receiving a teaspoon of pasteurized raw whole egg powder (active group) or placebo (control group) from 4 to 8 months of age (42) (Table 1). At 12 months of age, egg allergy incidence was not significantly different between groups; however, 31% of active group patients stopped egg powder ingestion due to allergic reactions (42).

The HEAP RCT included no-sensitized to HE infants who were randomized to receive pasteurized egg white powder (cases) or placebo (controls) from age 4–6 months until 12 months (43) (Table 1). At 12 months of age, sensitized or allergic subjects were not significantly different between cases and controls (43) (Table 1).

The STEP RCT included infants with a family history for allergy and without eczema, who were randomized to receive pasteurized raw whole egg or placebo from age 4 to 6.5 until 10 months (44) (Table 1). At 12 months of age, there was no significant difference between groups in the percentage of infants with IgE-mediated egg allergy (44) (Table 1).

Finally, the BEAT DBRCT included no-sensitized to HE infants with a family history of allergy who were randomized to receive whole-egg powder or placebo from age 4 to 6 until 8 months (45) (Table 1). At 12 months of age, there were no differences in egg allergy prevalence between groups. However, authors highlighted that early exposure to whole egg reduced sensitization to egg and induced egg-specific IgG4 production in high-risk infants (45).

### Peanut

Peanut allergy (PA), although lower in prevalence compared to CM and HE allergy, is burdened by a higher prevalence of severe allergic reactions and persistency over time. In infants affected by PA, a 10-fold higher environmental exposure to peanut was reported during the first year of life compared to atopic infants without PA (46). Available trials produced good evidence on a probable role of early peanut introduction in reducing the risk of PA in high-risk infants.

In the per-protocol analysis of the EAT trial, PA prevalence was significantly lower in the early-introduction group than in the standard-introduction group and the consumption of at least 2 g per week of peanut protein was associated with a significantly lower prevalence of PA than less consumption (39). These results should be interpreted in the light of a rate of adherence for peanut assumption of 61.9% (39).

The *Learning Early About Peanut Allergy* (LEAP) RCT evaluated which approach between early peanut introduction and avoidance diet was the most efficacious in PA prevention in infants at high risk for allergy (47). Specifically, infants with severe eczema and/or egg allergy were randomized in an avoidance group and an active group, consuming at least 6 g of peanut protein a week through peanut products for at least 3 time a week, from age 4–11 months (median 7.8 months, interquartile range 6.3–9.1) until 60 months (47). In the intention-to-treat evaluation, PA prevalence at 60 months of age, documented by an oral food challenge (OFC), was significantly lower in the active group compared to the avoidance group, independently from the sensitization or not of active group infants at baseline (on the basis of a skin prick test and specific IgE levels). Moreover, children belonging to the avoidance group had a significantly higher levels of peanut-specific IgE and lower levels of peanut-specific IgG4 than active group children (47). Results of this study pointed out important considerations concerning the continuous adaptation and evolution of immune system response. In particular, the efficacy of peanut introduction at a median age of 7.8 months in preventing PA suggested that the immunotolerance window is not likely limited between 4 and 6 months of age. Moreover, the higher significant difference of PA incidence at 5 years of age between the active group and the placebo group (3 vs. 17%) suggested the possibility of a progressive adaptation of the immune system, differently by studies evaluating CM and HE prevalence at 12 months of age. These design peculiarities could justify the better outcome reported in LEAP RCT compared to those of the CTs evaluating CM and HE early introduction.

In the LEAP-On study, 88.5% of participants in both groups from the LEAP study were instructed to avoid peanut consumption for 12 months (48). Authors demonstrated a significantly lower PA prevalence in children who had been in the active group during the LEAP study compared to children who had been assigned to the avoidance group (48), suggesting the beneficial role of peanut early introduction to achieve sustained tolerance despite the 12-month avoidance in the peanut consumption group children.

These findings significantly stimulated the development of Addendum Guidelines to specifically address the prevention of peanut allergy (35).

### Cereals and Fish

An observational study, carried out in infants from birth to a mean age of 4.7 years, prospectively evaluated the association between the timing of first exposure to cereals (oats, wheat, barley, or rye) and the development of wheat allergy based on parent report (49). At the end of follow-up, 1% of children were defined as having a wheat allergy. The probability to report a wheat allergy was increased 4-fold in children who first introduced cereals after 6 months of age compared to the ones who consumed cereals before 6 months of age, after controlling for family history of allergic diseases and history of food allergy before 6 months of age (49). Moreover, having a history of other food allergy before 6 months of age or a family history of allergic diseases (asthma, eczema, or hives) in a first-degree relative was independently associated with an increased risk of developing wheat allergy (49). Authors concluded that delayed first exposure to cereals after 6 months of age has not demonstrated a protective role in wheat allergy prevention but, on the contrary, could increase the risk of wheat allergy development (49). In another large observational Finnish birth cohort, introduction of wheat, rye, oats, or barley at 5–5.5 months, as well as introduction of fish at 6–9 months, has been related to a likely reduced risk of having asthma, allergic rhinitis, and atopic sensitization at 5 years of age, documented by a validated questionnaire (50).

With regard to fish allergy, an observational study, prospectively following infants from birth to 4 years of age, documented that regular fish consumption before 12 months of age was associated with a reduced risk of allergic disease and sensitization at 4 years of age (51). Consistently, results from another meta-analysis, aiming to specifically clarify the role of fish intake on different asthma outcomes in children, demonstrated that early introduction (between 6 and 9 months of age) and regular consumption of fish (at least once a week) decreased the risk, prevalence, and symptoms of asthma in children up to 14 years of age (52). Conversely, a recent meta-analysis reported both low-certainty evidence of association between fish introduction before age 6–12 months and reduction of allergic rhinitis, and very low-certainty evidence between fish introduction before age 6–9 months and reduction of allergic sensitization (53).

These findings should be interpreted considering the limitation due to the observational design of the studies that cannot demonstrate a specific cause–effect linkage between the timing of first ingestion of allergen and allergic symptoms.

EAT RCT also evaluated the effect of early introduction (before 6 months of age) of white-fish and wheat in FA prevention (39). In the per-protocol analysis, fish allergy rate was not significantly higher in the early-introduction group compared to the standard-introduction group, while there were no cases of wheat allergy in either group (39).

## Current Status for Non-IgE-Mediated Food Allergy

**Figure 1:**
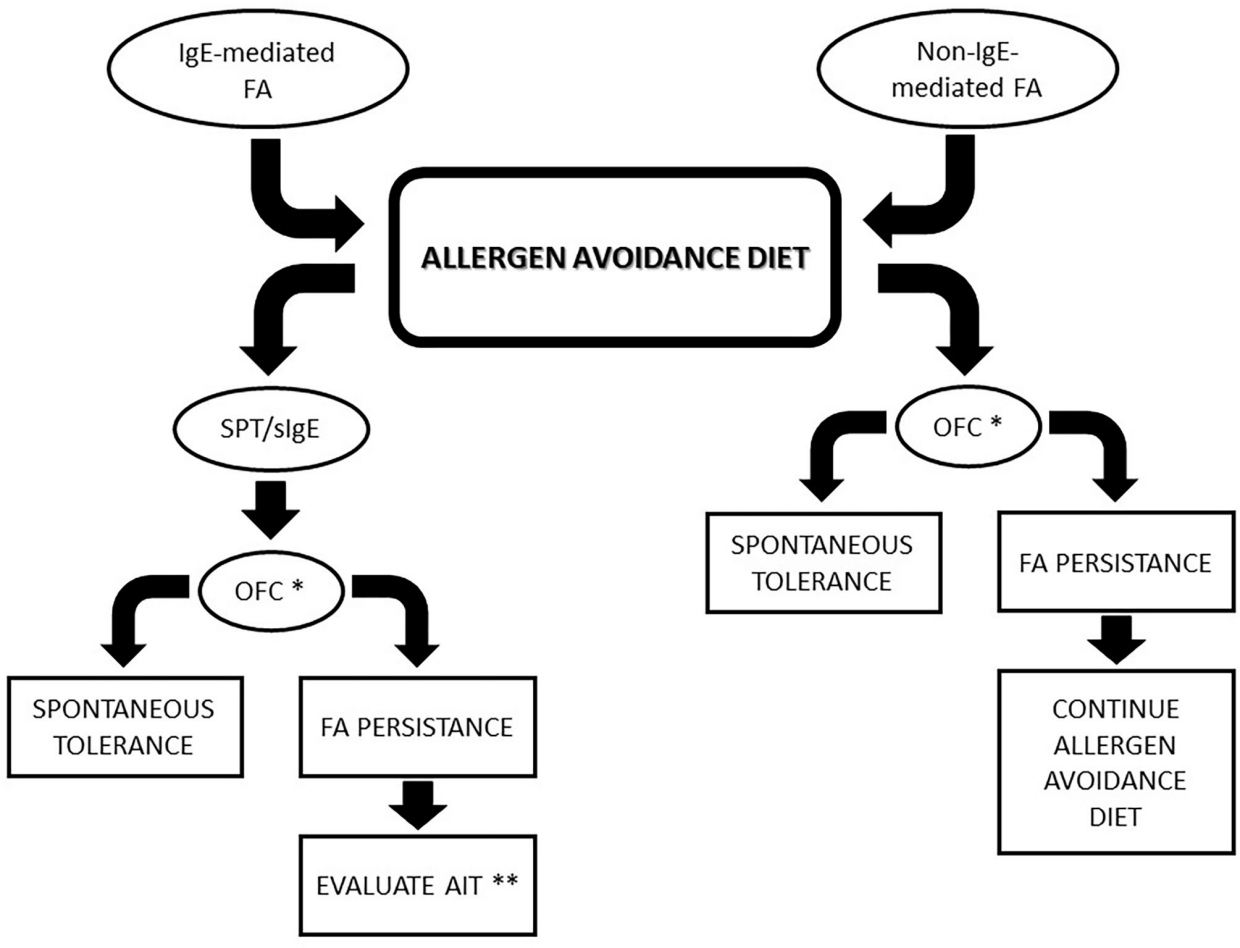
Algorithm for food allergies management. * In case of previous life-threatening reactions, specifically evaluate every patient before considering OFC. ** Consider AIT in children with IgE-mediated FA who did not achieve spontaneous tolerance at 4–5 years of age. FA, Food allergy; AIT, active immunotherapy; OFC, oral food challenge; SPT, skin prick test.

Recently, an increasing interest in diagnosis and management of non-IgE mediated FA has been developed. Typically, non-IgE-mediated FA has a delayed onset of allergic symptoms after culprit food ingestion. Non-IgE-mediated FAs include a wide range of disorders principally characterized by gastrointestinal symptoms. Management of non-IgE-mediated FAs is based on avoidance of the suspected trigger food and support to prevent nutritional deficiencies (Figure 1).

We focused on the nutritional and avoidance approach in food protein-induced enterocolitis syndrome (FPIES), food protein-induced allergic proctocolitis (FPIAP) and food protein-induced enteropathy (FPE), while eosinophilic esophagitis will not be discussed in this review.

CM and soy are the main allergens involved in non-IgE-mediated FAs, although they could be induced by rice, oats, and other foods (barley, chicken, turkey, egg, peanut, vegetables, fish, and mollusks) in relation to genetic, epigenetic, and environmental (e.g., age of introduction of the specific food into the diet) factors.

### FPIES

In the last decade, the clinical pattern, diagnosis, management, and natural history of FPIES have been extensively investigated (54). Clinically, in the majority of patients, FPIES is characterized by acute symptom onset, including repetitive protracted vomiting, ~1–4 h after trigger food ingestion, usually associated with pallor and lethargy and sometimes with watery diarrhea within 5 up to 24 h. Severe cases progress to hypothermia, methemoglobinemia, acidemia, dehydration, hypotension, and shock. Rarely, FPIES has a chronic trend associated with failure to thrive (FTT). The diagnosis is primarily based on the typical clinical picture and symptom resolution by avoidance diet. OFC, performed in a specialized setting, could be necessary in those cases with unclear clinical history (54). The first-line approach in FPIES treatment is a strict avoidance diet to offending trigger foods; however, long-term management should be tailored for every patient. Dietary management of FPIES follows empirical recommendation based on trigger food, possible cross-reactions with other food, and nutritional needs (55, 56). Breastfeeding should be recommended and maternal dietary elimination of trigger foods should not be routinely advised except for those cases in which allergic reaction occurs after breastfeeding, and it is associated to FTT in exclusively breast-fed infants (54, 57). In non-breast-fed infants with CM/soy-induced FPIES, an extensively hydrolyzed formula or amino acid-based formula (required in about 20% of patients) has been indicated (58, 59). Soy formula is not recommended before 6 months of age in CM-induced FPIES infants, whereas it may be thereafter considered in weaned infants, *vice versa* in soy-induce FPIES infants, even though a possible cross-reactivity between allergens has to be always considered (57, 58). In CM-induced FPIES infants, other animal milks (e.g., sheep and goat milk) should be avoided because of possible cross-reactions and insufficient nutritional value. Culprit food should be avoided also in baked and processed forms, unless baked products are already included in the diet, based on the assumption that high temperature does not destroy sequential allergenic epitopes recognized by T-lymphocytes (56). However, conclusive data evaluating baked food tolerance in FPIES are not available (55, 58). Due to the latency of symptoms onset and to possible reduction of required trigger dose in repeated episodes, it is difficult to establish a threshold dose able to provoke allergic reaction. This could partially justify the difficulty to plan an oral immunotherapy approach of FPIES (55).

Up to 80% of FPIES children have a single food allergy; therefore, delayed introduction of complementary food over 6 months of age is not recommended because of FPIES (10). Clinicians play a pivotal role to promote implementation of normal dietary variety, to prevent unnecessary avoidance, and to regularly monitor children growth.

The natural history of FPIES varies principally according to food trigger, feeding habits, and coexistence of IgE-mediated allergy; therefore, the timing for OFC to evaluate achieved tolerance varies accordingly. Conventionally, OFC is advised within 12–18 months after the most recent allergic reaction (56). Most FPIES patients achieve tolerance spontaneously within 5 years of age. CM tolerance has been reported in up to 85% of children by 3 years of age (57), whereas the average reported ages of tolerance are 12, 35, and 42 months for soy, grains and other solid foods, respectively (55, 58). However, these data do not derive from studies specifically designed to evaluate tolerance achievement; therefore, they may be biased by other factors.

### FPIAP

FPIAP is one of the most frequent causes of rectal bleeding in healthy infants, which histologically appears as an eosinophilic colitis (60–62). In healthy formula-fed infants with bloody-streaked stools, FPIAP is estimated to occur in up to 60%, while it is reported in up to 10% of extensively hydrolyzed-fed infants, although prevalence is not exactly determined (61, 63). FPIAP is a benign and self-limiting disease, associated to an excellent prognosis that starts in the first 2 weeks to 6 months of life and spontaneously resolves within 12 months of life in the majority of patients (64). FTT is not a peculiarity of FPIAP, while diarrhea, vomiting, abdominal pain, and anorexia could be present. CM is the most commonly involved trigger food. FPIAP onset is usually insidious, and it could depend on the timing of introduction of allergens in diet, even though up to 60% develop during exclusive breastfeeding. Culprit food elimination from child and mother diet usually determines symptom improvement in infants within 3 days (62). However, up to 20% of breastfed infants have a spontaneous resolution of bloody stools without the mother's avoidance diet; therefore, avoidance diet is not univocally advised in FPAIP management (65). In formula-fed infants, an extensive hydrolysate formula may be necessary (65). Jang et al. reported a failure of elimination diets in determining rectal bleeding resolution in 20% of infants of their cohort with histologic findings consistent with FPIAP (66). Arvola et al. assessed the effect of a CM-elimination diet on rectal bleeding duration in 40 infants, prevalently breast-fed (68%), with age between 4 weeks and 6 months (67). They randomized infants to the CM-elimination diet (amino acid-derived formula or CM-elimination diet in lactating mothers) or non-elimination diet for 1 month. CM IgE-mediated allergy was documented in 18% of infants by an OFC. Authors demonstrated that a CM-elimination diet did not significantly affect the duration or severity of rectal bleeding during follow-up; however, elimination diet seemed to shorten the duration of rectal bleeding in infants with CM IgE-mediated allergy (67). Therefore, after a limited period of CM-free diet, they suggested to perform a CM challenge, preceded by skin prick test (SPT) to CM proteins, to confirm diagnosis in those infants who had symptom resolution during the avoidance diet (67).

In a prospective population-based study, Elizur et al. evaluated the outcome of CM-free diet in 21 infants with rectal bleeding (19% exclusively breast-fed) (61). CM-free diet was carried out for a mean of 3 months, followed by the recovery of diet consumed before the initiation of rectal bleeding. All but one infant experienced a resolution of symptoms during CM-free diet. Also these authors highlighted that CM protein reintroduction, following symptom resolution, is often well-tolerated and is recommended to confirm the diagnosis and to avoid a prolonged unnecessary elimination diet (61). Moreover, this approach may be reinforced by evidence of negative effect of elimination diet on the possible switching toward CM IgE-mediated allergy (37).

Miceli Sopo et al. proposed a specific approach for infants with suspected FPIAP based on available evidence. In particular, they suggested waiting for a spontaneous resolution without an elimination diet in case of rectal bleeding ≤1 month, or starting an CM elimination diet in case of rectal bleeding >1 month, followed by CM challenge if rectal bleeding disappears (65). If hematochezia returns, these authors suggested restarting the elimination diet for 3 months further and to perform specific SPT before the next CM challenge (65).

### FPE

FPE starts in the first year of life, a few weeks after the introduction of an allergen, and it resolves within 2 years of age in the majority of patients. Protracted diarrhea is the typical onset symptom and could be associated with abdominal pain and distension, early satiety, emesis, malabsorption with steatorrhea, and FTT (62, 68). The clinical picture of FPE could resemble a post-enteritis syndrome; however, FPE could effectively develop after an infectious gastroenteritis (62). Histologically, FPE is characterized by lymph nodular hyperplasia in the duodenal bulb and villous structure alterations (68). CM is the most common trigger, but soy, rice, poultry, fish, and shellfish have also been reported as triggers. FPE has not been reported in exclusively breastfed infants (64).

Data on FPE nutritional management are lacking. Avoidance of culprit food determines the resolution of symptoms in 1–3 weeks, and the association of extensively hydrolyzed formula has been suggested (64). In cases with malabsorption and FTT, parenteral nutrition may be necessary (68).

## Looking at Tolerance

Tolerance is considered the achievement of a goal to safely consume a normal serving of food containing the trigger allergen, previously counted harmful, despite a period of absence of exposure (8). Tolerance could be spontaneously achieved as in the majority of children affected by CM, HE, wheat, and soy protein IgE-mediated allergy. On the other hand, patients allergic to peanut, tree nut, and fish have a natural history of allergy that is quite disappointing with persistence of symptoms. Persistence of allergy could negatively affect the QoL of children and their family. Therefore, patients with FA should be regularly followed up to avoid an inappropriate or unnecessarily prolonged elimination diet that possibly conditions social life, dietary nutritional intakes, and growth. In this perspective, a specific management for every patient should be programmed. Specific IgE testing (*in vitro* or SPT) could be the first step to assess allergen sensitization decrease and to address subsequent steps, since decreasing specific IgE testing response over time seems to be related with clinical tolerance; however, specific IgE testing has limited value in guiding the timing of OFC.

OFC is able to demonstrate an achievement of tolerance and it should be performed at regular intervals, based on clinical patient history, trigger allergen, SPT, and/or specific IgE results. Currently, re-testing OFC has been suggested every 6–12 months from the last allergic reaction in patients with CM or HE allergy and every 2 years in cases of peanut and tree nut allergy in the absence of reaction due to accidental ingestion of trigger food (10).

On the way toward tolerance, it has been demonstrated that extensively heated allergens are tolerated earlier than raw allergens in IgE-mediated HE allergy and, to some extent, CM allergy. In these patients, introduction of baked products containing CM and HE proteins is supported by current evidence because it is safe, convenient, and well-accepted by patients, and it seems to accelerate tolerance achievement of raw allergens (69).

In recent years, AIT became an increasingly important therapeutic strategy to approach FA, potentially able to induce improvement through desensitization to a specific food (70). AIT is indicated in patients with confirmed diagnosis of IgE-mediated FA in whom spontaneous tolerance did not develop, and an avoidance approach is ineffective or causes severe limitations to a patient's QoL (8) (Figure 1). Currently, it is recommended to wait for the acquisition of tolerance until about 4–5 years of age before considering AIT, although a clinical picture of every patient will be specifically evaluated.

AIT is able to increase the threshold of trigger food intake amount, reducing allergic symptoms and the incidence of life-threatening reactions, and, in selected patients, to attain post-desensitization effectiveness (8). In light of available evidence, desensitization is a more feasible objective. In that condition, allergic symptoms may occur, with the same characteristics or attenuated, when administration of AIT is interrupted.

AIT is burdened by side effects, including systemic and potentially life-threatening reactions. In addition, it is usually a long-term treatment, carried out at a specialized center. Therefore, patients who underwent AIT should be carefully selected.

The most common approaches for AIT is the oral immunotherapy (OIT), consisting in oral administration and prompt ingestion of food allergen. The use of sublingual immunotherapy (SLIT), subcutaneous immunotherapy (SCIT), and epicutaneous immunotherapy (EPIT) is less common.

A meta-analysis of RCT and non-randomized controlled trials (CTs) evaluating different allergens documented that both OIT and SLIT are efficacious in terms of desensitization in children. Based on the results of RCTs, this meta-analysis suggested, but did not confirm, post-desensitization effectiveness (or sustained unresponsiveness) in children treated with OIT (71).

More recently, in a phase 3 trial, carried out in subjects who were highly allergic to peanut, OIT with AR101 (a peanut-derived investigational biologic oral immunotherapy drug) resulted in desensitization in children and adolescents (72). In particular, a significantly higher percentage of active group patients were able to ingest a single dose of at least 600 mg of peanut protein (cumulative dose ≥ 1043 mg) during the exit food challenge, with no dose-limiting symptoms and lower symptom severity, compared to the placebo group, although desensitization was evaluated after only 6 months of maintenance regimen (72).

Interestingly, OIT has been evaluated in subjects affected by tree nut allergy, a condition characterized by a very frequent cross-reaction between allergens (about 86% of children with a tree nut allergy develop sensitization to another tree nut by adolescence) that are rarely outgrown spontaneously, that is burdened by a high risk of life-threatening reactions and often heavily and negatively affects the QoL of patients and their family, for which no CTs on the role of early allergen introduction in tree nut allergy prevention have been conducted. Particularly, in a preliminary, prospective cohort study involving subjects with tree nut co-allergies, OIT to walnut resulted in desensitization to walnut as well as cross-desensitization to other tree nuts, with a reasonable safety profile (73). This promising result supports the possibility to simultaneously induce desensitization to cross-reactive allergens by OIT, but it should be confirmed by RCT in a larger cohort of patients (73).

OIT is usually carried out with fresh or natural raw food or, alternatively, with different processed foods in those patients with a history of severe allergic reactions. However, the concentration of an allergen and, consequently, its allergenic potential, changes in relation to food form (raw, cooked, or processed food) and type of processing (homogenization, hydrolysis, irradiation, etc.) in case of processed foods.

Many issues are still unsolved in the management of AIT, including the standardization of food and shared protocol(s) employed in OIT, identification of predictive biomarkers, and long-term effectiveness (permanent tolerance).

## Conclusions

In summary, delayed food introduction as well as assumption of allergenic foods before 4 months of age has been proven ineffective in FA prevention, while early introduction of potential trigger food, between 4 and 6 months of age, has been suggested as a preventive strategy for FA, although reliable evidence is available only for peanut allergy. The current approach to FPIES, FPIAP, and FPE is based on elimination diet and nutritional counseling; however, strong supporting evidence on dietary management are lacking. Key points of FA discussed above are outlined in Table 2.

**Table 2:** Key points.

		**Key points**
IgE-mediated FA	Milk	The effectiveness of CM early introduction in allergy prevention has not been demonstrated by the only one interventional study evaluating CM early introduction.
	Egg	Although two interventional studies suggested a positive effect of the early introduction strategy for HE allergy prevention, the other four did not confirm this result.
	Peanut	Two interventional studies suggested a positive effect of peanut early introduction in PA prevention.
	Cereal and fish	Effectiveness of early introduction in fish allergy prevention has not been demonstrated by the only one interventional study evaluating fish early introduction. In the same study, no cases of wheat allergy were reported.
Non-IgE-mediated FA	FPIES	Currently, the first-line approach is the strict avoidance diet of offending foods, followed by periodic re-evaluation of tolerance achievement by OFC, according to allergen and patient clinical history.
	FPIAP	Avoidance diet should not be univocally advised in breastfed infants with FPIAP and their mothers; spontaneous resolution of symptoms could be expected if rectal bleeding has lasted <1 month.
	FPE	Avoidance of culprit food, sometimes associated to extensively hydrolyzed formula, determines the rapid resolution of symptoms.

*FA, Food allergy; CM, cow's milk; HE, hen's egg; PA, peanut allergy; OFC, oral food challenge; FPIES, food protein induced enterocolitis syndrome; FPIAP, food protein–induced allergic proctocolitis; and FPE, food protein–induced enteropathy*.

In this context, clinicians play a central role in promoting dietary variety and preventing unnecessary or prolonged avoidance diet in children affected by FA.

As suggested by the results of the most recent CTs, several aspects, including study design, age of allergen introduction, quantity and form of assumed food (e.g., raw or cooked food), have to be considered in outcome interpretation, since they can affect the effectiveness of early introduction strategy for FA prevention. Based on available data, we recommend, both in infants at high-risk for allergies and at normal-risk, to begin the introduction of complementary foods between 4 and 6 months of age, continuing progressively with different foods, in accordance with infant development and familial and cultural customs, carrying on breastfeeding up to 6 months or beyond.

Infants who have been diagnosed with FA and/or are affected by atopic dermatitis with positive SPT to a specific food should undergo OFC under medical supervision in a specialized center to evaluate the achievement of spontaneous tolerance before reintroducing the culprit food into the diet.

In patients with persistent IgE-mediated FA, in which elimination diet represents often a heavy therapeutic option and, accidental exposure could provoke severe adverse reactions, AIT represents an emerging reality potentially able to actively treat FA inducing desensitization.

## Author Contributions

GP conceived, reviewed, and revised the manuscript. DC drafted and wrote the manuscript. TA and MW were involved in literature search and revised the manuscript. LC and FL prepared the table and the figure. All authors approved the final manuscript as submitted and agreed to be accountable for all aspects of the work.

## Conflict of Interest

The authors declare that the research was conducted in the absence of any commercial or financial relationships that could be construed as a potential conflict of interest.

## Abbreviations

FA: Food allergy
AIT: active immunotherapy
QoL: quality of life
CM: cow's milk
HE: hen's Egg
WHO: World Health Organization
DBRCT: double-blind randomized controlled trial
EAT: Enquiring About Tolerance
PETIT: Prevention of Egg Allergy with Tiny Amount Intake
STAR: Solid Timing for Allergy Research
HEAP: Hen's Egg Allergy Prevention
STEP: Study Starting Time of Egg Protein
BEAT: Beating Egg Allergy Trial
PA: peanut allergy
LEAP: *Learning Early About Peanut Allergy*
OFC: oral food challenge
FPIES: food protein induced enterocolitis syndrome
FPIAP: food protein–induced allergic proctocolitis
and FPE: food protein–induced enteropathy
FTT: failure to thrive
SPT: skin prick test
OIT: oral immunotherapy
SLIT: sublingual immunotherapy
SCIT: subcutaneous immunotherapy
EPIT: epicutaneous immunotherapy
CTs: controlled trials.
